# Dramatic Intracerebral Hemorrhagic Presentations of Reversible Cerebral Vasoconstriction Syndrome: Three Cases and a Literature Review

**DOI:** 10.1155/2014/782028

**Published:** 2014-01-12

**Authors:** Joel M. Stary, Bonnie H. Wang, Seong-Jin Moon, Huan Wang

**Affiliations:** ^1^Department of Neurosurgery, Virginia Commonwealth University School of Medicine, Richmond, VA 23298, USA; ^2^Department of Internal Medicine, University of Illinois College of Medicine at Urbana-Champaign, Urbana, IL 61801, USA; ^3^University of Illinois College of Medicine at Urbana-Champaign, Urbana, IL 61801, USA; ^4^Department of Neurosurgery, University of Illinois College of Medicine at Urbana-Champaign, Carle Foundation Hospital, 602 West University Avenue, Urbana, IL 61801, USA

## Abstract

Reversible cerebral vasoconstriction syndrome (RCVS) refers to a number of disorders characterized by severe and sudden-onset (“thunderclap”) headaches and angiographic features of reversible, segmental, multifocal vasoconstriction of cerebral arteries. Although RCVS generally resolves without significant sequelae, a rare and possibly underrecognized hemorrhagic presentation has a worse potential course. We report three cases of hemorrhagic RCVS and review the literature. Three females (42, 54, and 33 years old, resp.) presented with severe headache, neurological deficits, and dramatic intracerebral hemorrhage (ICH). Patient 1 presented comatose with a 9 × 4 × 6.6 cm left deep intraparenchymal hemorrhage (IPH) and 1 cm midline shift. She underwent emergent surgical intervention. Patient 2 had a 3.3 × 1.5 cm left superior frontal IPH that enlarged to 4 × 2.5 cm within 12 hours with worsening headache and neurological deficits. She was successfully managed nonoperatively. Patient 3, after uncomplicated pregnancy and delivery, presented with a 1.5 cm left superior parietal IPH on postpartum day 7. Two days later, she acutely developed right hemiplegia. Repeat CT demonstrated a new 3.3 × 1.7 cm left frontal IPH. She was also successfully managed nonoperatively. Many diverse conditions are grouped within the category of RCVS. Dramatic ICH remains a rare and possibly underrecognized presenting feature. Prompt diagnosis and management are essential for obtaining the best outcome.

## 1. Introduction

Reversible cerebral vasoconstriction syndrome (RCVS) refers to a number of disorders characterized by severe and sudden-onset (“thunderclap”) headaches and the angiographic feature of segmental, multifocal vasoconstriction of cerebral arteries that resolves within 12 weeks of presentation [1, 2]. Presentation can include focal neurological deficits, nausea, photophobia, and/or seizures; however, there is no exclusive cardinal feature specific to RCVS. Due to the necessity of a follow-up diagnostic angiogram, the diagnosis during acute presentation is principally one of exclusion.

Many factors have been tied to the onset of RCVS, including the postpartum period, exposure to blood products, exposure to vasoactive drugs, migraines, hypertension, neoplasms, trauma, and increased intracranial pressure due to bending over, coitus, or valsalva [1, 2]. The diversity of possible triggering conditions and presentations means nearly every clinical setting can encounter RCVS, and those varied specialty encounters have led to numerous designations throughout the years, including thunderclap headache with reversible vasospasm [3], migrainous vasospasm [4], migrainous angiitis [5], benign angiopathy of the central nervous system [6, 7], CNS pseudovasculitis [8], isolated benign cerebral vasculitis [9], Call or Call-Fleming syndrome [10], postpartum angiopathy [11], and drug-induced cerebral arteritis [12]. Considerable work has sought to align these varied designations under the banner of RCVS, thus elucidating the ties between the aforementioned conditions and providing some insight into its course [13].

RCVS is historically a benign condition; its presentation thought to mimic more serious conditions like subarachnoid hemorrhage (SAH). However, awareness of the condition has grown and angiography is now standard in a variety of clinical workups. As more angiograms are being performed, RCVS is identified more frequently and is being associated with far less benign presentations. In particular, the hemorrhagic presentation is now increasingly recognized and is associated with a potentially more sinister course, indicating the need for prompt diagnosis and treatment [14, 15]. Dramatic intracerebral hemorrhage (ICH) remains a rare and possibly underrecognized presenting feature of RCVS. In this light, we report three such cases and review the current data, theories of etiology, and treatment strategies for RCVS with hemorrhagic presentation.

## 2. Case Reports

### 2.1. Case  1

**Figure 1:**
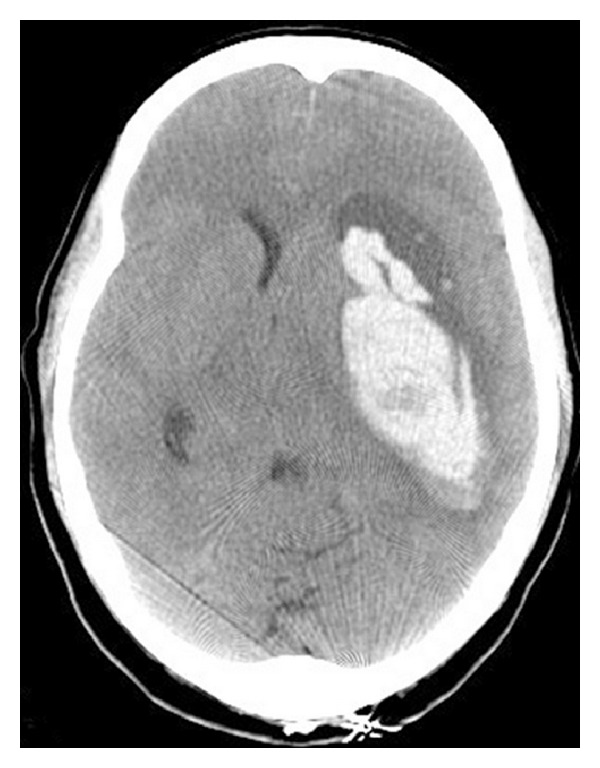
Cranial CT without contrast demonstrating a 9 × 4 × 6.6 cm left deep intraparenchymal hemorrhage without any associated SAH, with 1 cm midline shift.

**Figure 2:**
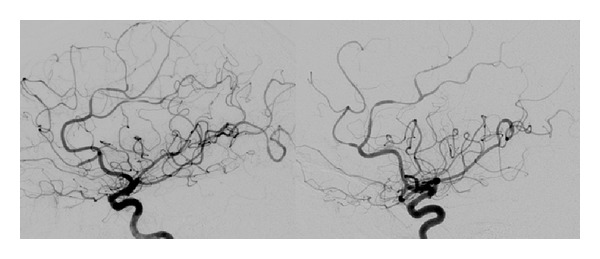
Bilateral internal carotid artery angiography, lateral view, demonstrating severe multifocal vasoconstriction but without any evidence of arteriovenous shunting or aneurysms.

**Figure 3:**
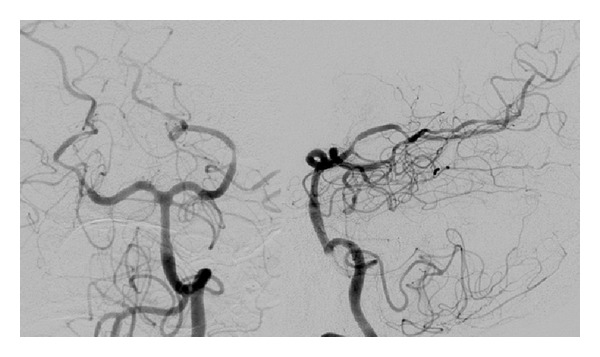
Left vertebral artery angiography, AP, and lateral views, demonstrating severe multifocal vasoconstriction but without any evidence of arteriovenous shunting or aneurysms.

A 42-year-old female with history of migraines experienced recurrent, sudden-onset, severe headaches for four days prior to her day of admission. On the day of admission, she developed headache after sexual intercourse and used her standard sumatriptan dose. Approximately four hours later, her partner noticed expressive aphasia and right-sided paresis. She worsened progressively and presented to the ED in a comatose state. Initial CT showed a 9 × 4 × 6.6 cm left deep intraparenchymal hemorrhage (IPH), left lateral ventricle compression, and 1 cm midline shift without any associated SAH (Figure 1). She underwent emergent left decompressive craniectomy, with hematoma evacuation and ventriculostomy placement. Cerebral angiogram demonstrated severe multifocal vasoconstriction but was without any evidence of arteriovenous shunting or aneurysms (Figures 2 and 3). A one-week follow-up angiogram documented improvement of vasoconstriction and the three-month follow-up angiogram demonstrated complete resolution. Extensive workups for vasculitic, immunologic, and infectious causes were negative along with essentially normal CSF analysis and leptomeningeal biopsy results. Cranial MRI evaluations did not demonstrate any unexpected findings. She made significant neurological improvement with a Modified Rankin Scale of 3 at three months.

Review of this patient's chart for additional potential contributing factors revealed a long-standing history of migraines treated with sumatriptan, several weeks' use of over-the-counter ephedra for weight loss, and admission BP >180/90 mm Hg.

### 2.2. Case  2

**Figure 4:**
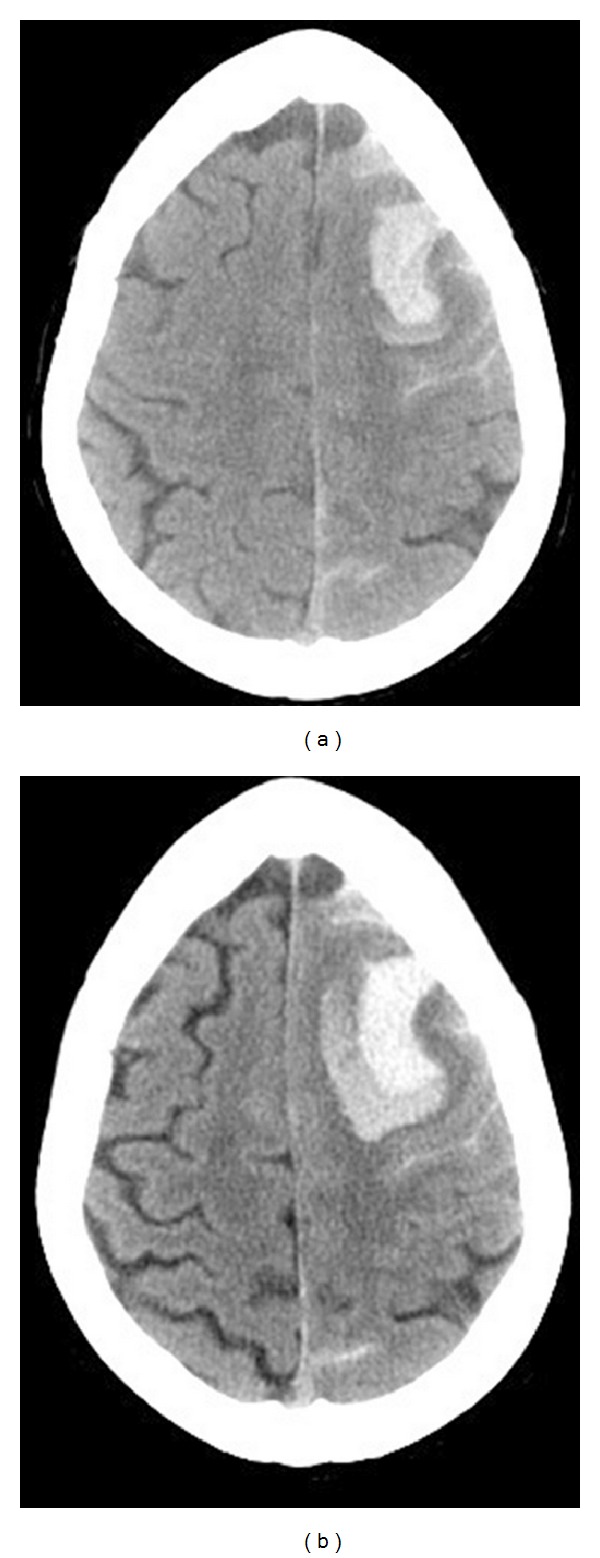
Initial cranial CT without contrast (a) and CT 12 hours later (b). A primary left superior frontal hematoma (3.3 × 1.5 cm) with minimal subarachnoid hemorrhage (a); significant hematoma expansion (4 × 2.5 cm) is evident on the repeat CT (b).

A 54-year-old female presented to the ED with five-hour history of severe headache, nausea, mild expressive aphasia, acalculia, and mild perseveration. She experienced acute onset of the headache and nausea, which occurred specifically while bending over to pick up her grandchild. Headache and nausea persisted and the additional neurological findings manifested over the next several hours. Initial CT demonstrated a 3.3 × 1.5 cm left superior frontal IPH with 2 mm midline shift and minimal adjacent subarachnoid component (Figure 4(a)). Twelve hours later, she experienced increasing headache and worsening neurological deficits. A repeat CT demonstrated interval hematoma expansion, now measuring approximately 4 × 2.5 cm (Figure 4(b)). The subsequent cerebral angiogram demonstrated severe multifocal vasoconstriction without any evidence of arteriovenous shunting or aneurysms. Although she was successfully managed nonoperatively and was making an excellent neurological recovery, one week follow-up angiogram documented worsening of vasoconstriction. The three-month follow-up angiogram demonstrated complete resolution. Extensive workups for vasculitic, immunologic, and infectious causes were negative along with essentially normal CSF analysis and leptomeningeal biopsy results. Cranial MRI evaluations did not demonstrate any unexpected findings. She achieved complete neurological recovery with a Modified Rankin Scale of 0 at three months.

Review of this patient's chart for additional potential contributing factors revealed a long-standing history of hypertension treated with hydrochlorothiazide, an admission BP >150/80 mm Hg, and alcohol use approximated at four glasses of wine per night without binge episodes.

### 2.3. Case  3

**Figure 5:**
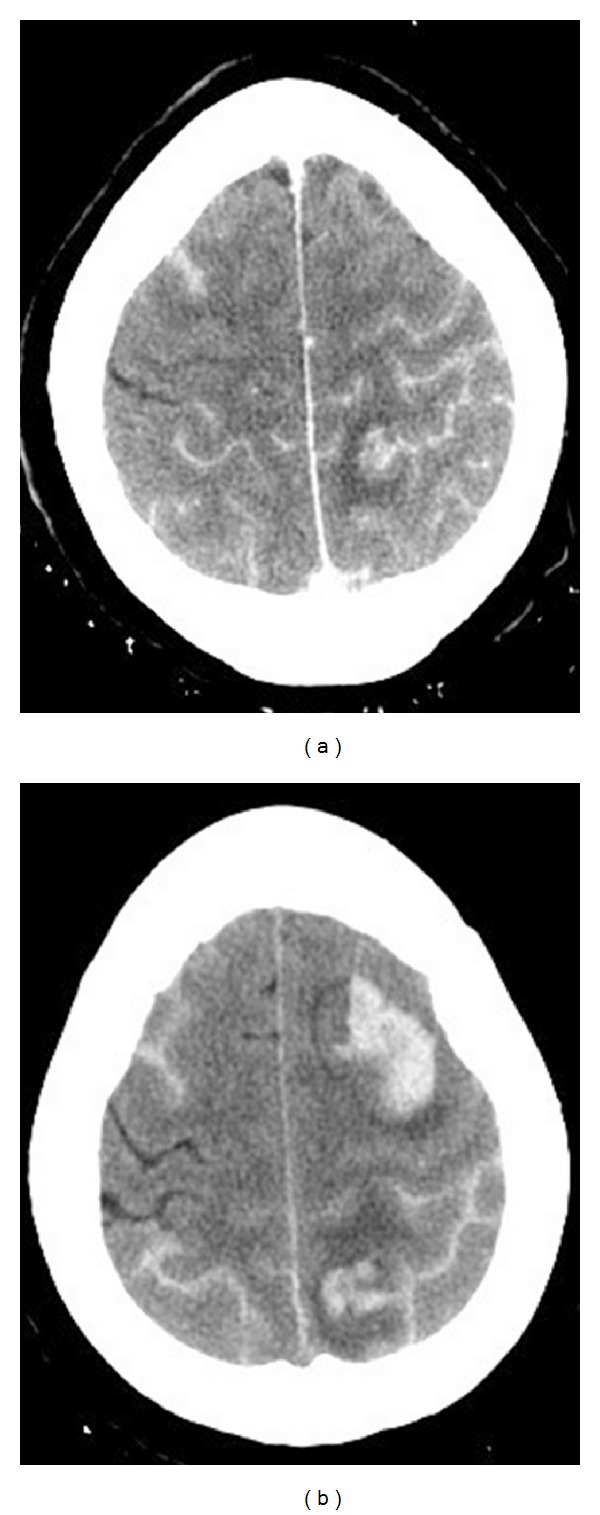
Initial cranial CT demonstrated convexity subarachnoid hemorrhage and a 1.5 cm left superior parietal acute intraparenchymal hemorrhage (a). Three days later, the patient developed acute right hemiplegia. Repeat CT demonstrating a new 3.3 × 1.7 cm left frontal intraparenchymal hemorrhage (b).

A 33-year-old female with a history of intermittent migraine headache for years experienced sudden-onset of severe headaches, projectile vomiting, and visual disturbance the evening prior to her day of admission. She delivered her third child vaginally 6 days earlier without complication, after an uneventful course of pregnancy. Initial CT demonstrated a 1.5 cm left superior parietal IPH and bilateral convexity subarachnoid hemorrhage (Figure 5(a)). Two days later, she developed acute right hemiplegia and repeat CT demonstrated a new 3.3 × 1.7 cm left frontal IPH (Figure 5(b)). The cerebral angiogram demonstrated severe multifocal vasoconstriction without any evidence of arteriovenous shunting, aneurysms, or venous sinus thrombosis. She was successfully managed nonoperatively. The three-month follow-up angiogram demonstrated complete resolution. Extensive workups for vasculitic, immunologic, and infectious causes were negative. Cranial MRI evaluations did not demonstrate any unexpected findings. She made excellent neurological recovery with a Modified Rankin Scale of 1 at three months.

Review of this patient's chart for additional potential contributing factors revealed history of mild to moderate intermittent migraine headache approximately every 1-2 months for years without prescription medication treatment. Her last episode of migraine headache was approximately 2 weeks before this event. Her admission BP was >150/80 mm Hg. Her pregnancy was uncomplicated with no infection, proteinuria, hypertension, or preeclampsia.

## 3. Discussion

This paper profiles three females (42, 54, and 33 years old) who presented with severe headache and neurological deficits. They were found to have large ICH and all had radiographic evidence of segmental, multifocal vasoconstriction. Patient 1 had a 9 cm left deep IPH with 1 cm midline shift and underwent emergent surgical intervention; patient 2 had a 3.3 × 1.5 cm left superior frontal IPH that enlarged to 4 × 2.5 cm within 12 hours with increasing headache and worsening neurological deficits, and she was successfully managed nonoperatively; patient 3 initially had a 1.5 cm left superior parietal IPH but developed acute right hemiplegia two days later with a new 3.3 × 1.7 cm left frontal IPH, and she was successfully managed nonoperatively. All patients' initial cerebral angiograms demonstrated diffuse, severe, short-segmental vasoconstriction in anterior and posterior circulations, indicating that the vasospasm was not likely hemorrhage-induced. Three-month follow-up angiograms demonstrated complete resolution of vasoconstriction in all three patients.

There were several other factors present. Patient 1 had several days of recurrent, sudden-onset migrainous headaches and a coitus-induced headache with subsequent triptan use hours before she developed neurological deficits. Patient 2 developed neurological deficits shortly after initial headache onset and experienced onset while bending over. Patient 3 developed severe headache and visual symptoms on postpartum day 7 after an uncomplicated course of pregnancy and delivery. Patients 1 and 2 had hypertension. Patients 1 and 3 had migraine headaches. Patients 2 and 3 had evidence of adjacent subarachnoid components. Early follow-up angiograms showed improvement of vasoconstriction in patient 1 but worsening vasoconstriction in patient 2. Finally, at three-month followup, the Modified Rankin Scale scores were 3 (patient 1), 0 (patient 2), and 1 (patient 3); however, all three had significant improvements from presentation.

No patients had any indications of aneurysm or other mass. For patients 1 and 2, CSF analyses were normal, extensive workups for vasculitic, immunologic, and infectious causes were negative, and leptomeningeal biopsy results were normal; CSF analysis and leptomeningeal biopsy were not performed for patient 3. For all 3 patients, cranial MRI did not demonstrate any unexpected findings. These patients shared two key risk factors for developing RCVS: sex and age.

### 3.1. RCVS

Reversible cerebral vasoconstriction syndrome is a constellation of disorders characterized by prolonged, segmental vasoconstriction of anterior and posterior circulation arteries. Vasoconstriction can be symptomatic, yet also can persist after resolution of symptoms. RCVS is most commonly reported in females in their 4th and 5th decades of life. Additional factors that may contribute to the etiology of RCVS include: hypertension, pregnancy, or the postpartum period and activities that increase intracranial pressure such as bending over [16], coitus [5, 17, 18], and the use of vasoactive substances like serotonergics [12, 19], triptans [20], ergot derivatives [21, 22], or sympathomimetics [23].

### 3.2. Hemorrhagic Manifestations

RCVS generally presents with a severe “thunderclap” headache [24], and cases generally resolve without significant sequelae. A patient's description of thunderclap headache or SAH can be remarkably similar (worst headache of my life), and RCVS has historically been considered the more benign etiology [25]. However, a meta-analysis of case studies [26] and a recent prospective study [14] show there may be a significant number of hemorrhagic manifestations either on presentation or within the 1st week of headache onset, lending a sinister bent to the syndrome's potential course and suggesting the need for emergent diagnosis and treatment. The prospective study also suggests that cortical subarachnoid hemorrhage is the most likely hemorrhagic outcome, a finding in line with other retrospective studies and individual case studies [27–36].

### 3.3. Intracerebral Hemorrhage

Compared to SAH, isolated ICH is a fairly rare potential complication of RCVS, but one that can result in significant long-term impairment or death [37, 38]. A large retrospective study from two hospitals' data found isolated ICH with a frequency of 6% in 139 patients [34]; prospective studies from two headache clinics and one emergency headache center showed isolated ICH frequencies of 0% [39], 0% [40] and 3% [14] in 52, 25, and 89 patients, respectively. In the retrospective study, all ICHs (with/without SAH and/or infarction) affected nearly 15% of patients with hemorrhagic outcomes. Ducros et al. [14] sought potential risk factors in their patient population and identified female gender and history of migraine as the only independent risk factors for hemorrhagic manifestation.

### 3.4. Risk Factors in Our Patients

Two of the three patients (patient 1 and patient 3) identified in this study had a history of migraine. Patient 1 required emergent surgical treatment and while improved greatly from presentation, still exhibited significant residual neurological deficits at three-month followup. Patient 3, although initially presented with severe headaches, visual symptoms, and a 1.5 cm left superior parietal IPH, developed acute right hemiplegia 2 days later with a new 3.3 × 1.7 cm left frontal IPH. At three-month followup, while improved greatly from presentation, she still exhibited residual neurological deficits. Patient 1 had a history significant for ephedra use, which is associated with RCVS and stroke [41, 42] and triptan use just before onset of neurological symptoms. The frequency with which she used her triptan for migraine relief is unknown, but ICH and vasospasm have been documented after overuse of antimigraine medications [20]. It is unclear how these drugs may affect its severity or progression, but their association with RCVS and hemorrhage warrants further investigation.

### 3.5. Pathophysiology

The pathophysiology of RCVS is not well understood. Due to the rarity of the condition, larger scale studies to investigate cause are relatively scarce and the knowledge base borrows heavily from the condition's similarity to and association with other vasoconstrictive disorders. Based on the known risk factors that affect vasoconstriction, current thought is that the condition is a result of an aberrant sympathetic response. This may be an induced alteration in response due to vasoactive drugs, an underlying inability to cope with activities that cause surges in intracranial pressure, and/or a systemic problem in people with hypertension.

SAHs can trigger cerebral vasospasm, but it is thought that RCVS and hemorrhage induced vasospasm are truly different processes because RCVS demonstrates vasoconstriction that is not limited to the affected hemisphere, and the headache associated with RCVS resolves long before the resolution of vasoconstriction, whereas vasoconstriction in vasospasm is more commonly delayed from the onset of the hemorrhage, generally limited to the hemorrhage affected region, and the symptoms typically resolve with the resolution of vasoconstriction. Thus, the components of the vasoactive milieu responsible for post-SAH cerebral vasospasm, such as endothelin-1, serotonin, nitric oxide, prostaglandins, and catecholamines are less likely to be the underlying cause of RCVS [43]. The association with pregnancy (postpartum angiopathy) has led people to suggest connections between RCVS and preeclampsia. Indeed, hypertension is a major component of both diseases, and levels of placental growth factors, sFlt-1 and TGF-beta1, known to be elevated in eclampsia, have been demonstrated in patients with RCVS in the postpartum period.

At the genetic level, recent work has shown a significant association between brain-derived neurotrophic factor (BDNF) polymorphisms and the severity of vasoconstriction in people with RCVS [44]. Secreted BDNF is used by signaling pathways throughout the body, and it was shown that there are functional polymorphisms with differing levels of expression [45]. BDNF is hypothesized to have a role in the modulation of both the sympathetic and parasympathetic nervous systems [44, 46]. Kasselman et al. [47] demonstrated that in the presence of baseline sympathetic overactivity, increased levels of BDNF trigger perivascular inflammation and vasoconstriction. One of the polymorphisms with higher expression levels was then associated with higher levels of serum C-reactive protein and unstable angina [48], and it is this form that was associated with more severe vasoconstriction in patients with RCVS. Thus, a picture emerged wherein people with particular polymorphisms would not only have increased levels of BDNF, but also have subsequent dysregulation of their autonomic nervous systems that would increase their susceptibility for vasoconstriction. However, the tendency of this dysregulated state to produce perivascular inflammation, which is not seen in RCVS, suggests we do not have the full picture yet and more work needs to be done.

### 3.6. Treatment

Treatment modalities for RCVS are being explored. The signs and symptoms of RCVS can mimic those of readily assessed acute processes such as ICH or infarct but can also resemble those of more long-term processes such as primary angiitis of the central nervous system (PACNS). Differentiation of these chronic conditions requires some time, and during this intervening time, patients are often treated symptomatically, with a calcium channel blocker and/or with glucocorticoids. As of yet, no studies have closely examined the effectiveness of these therapies alone or combined, but a retrospective analysis shows that patients with glucocorticoid treatment trend toward poorer outcomes. Given the importance of time-to-treatment of steroids with PACNS and trend to harm if RCVS patients receive glucocorticoids, there should be careful investigation of the frame of onset before treating with steroids.

In regard to calcium channel blockers, there have been no prospective, randomized, placebo-controlled studies to investigate their efficacy. Nimodipine is reportedly the most widely used treatment, and while case series indicated there was a more rapid improvement in headache, there were apparently no other outcomes that were improved with nimodipine as compared to conservative, symptomatic treatment [34, 49]. Likewise, use of verapamil was associated with radiographic improvement but was not associated with improved clinical outcomes [50].

## 4. Conclusion

Our study identified three patients who presented to the ED with severe, sudden-onset headaches and neurological deficits. All had dramatic ICH, and two also demonstrated a subarachnoid component, with no evidence of ruptured or unruptured aneurysm or subdural hematoma. All had multiple risk factors for RCVS and segmental multifocal constriction of cerebral arteries was demonstrated by angiogram. The short-segment constrictions were seen in both hemispheres and affected both the anterior and posterior circulation, lessening the likelihood that the constrictions observed were hemorrhage-induced vasospasm. For all three patients, three-month follow-up angiograms demonstrated complete resolution of the vasoconstriction. Given the patient presentations and negative workups for immunologic, infectious, and vasculitic etiologies, it is most likely that these patients had RCVS with hemorrhagic manifestation, rather than other vasoconstriction syndromes [51, 52]. The three had varied clinical presentations, varied hospital courses, and the only common risk factors were their sex and age. These patients were all identified after hemorrhage had occurred.

Currently, many diverse conditions are grouped within the category of RCVS; yet, they may or may not share the same underlying pathophysiology. Although there is an increased awareness of hemorrhagic presentations of RCVS, dramatic ICH remains a rare and possibly underrecognized presenting feature of RCVS. Familiarity with this clinical entity for prompt diagnosis and management is essential for cerebrovascular neurosurgeons, vascular neurologists, neurocritical care physicians, and neurohospitalists to create the best possible patient outcome.

## Conflict of Interests

The authors declare that there is no conflict of interests regarding the publication of this paper.
